# Vector competence, vectorial capacity of *Nyssorhynchus darlingi* and the basic reproduction number of *Plasmodium vivax* in agricultural settlements in the Amazonian Region of Brazil

**DOI:** 10.1186/s12936-019-2753-7

**Published:** 2019-04-04

**Authors:** Maria Anice M. Sallum, Jan E. Conn, Eduardo S. Bergo, Gabriel Z. Laporta, Leonardo S. M. Chaves, Sara A. Bickersmith, Tatiane M. P. de Oliveira, Elder Augusto G. Figueira, Gilberto Moresco, Lêuda Olívêr, Claudio J. Struchiner, Laith Yakob, Eduardo Massad

**Affiliations:** 1 Departamento de Epidemiologia, Faculdade de Saúde Pública, Universidade de São Paulo, São Paulo, SP Brazil; 2 Wadsworth Center, New York State Department of Health, Albany, NY USA; 3 Department of Biomedical Sciences, School of Public Health, State University of New York, Albany, NY USA; 4 Superintendência de Controle de Endemias, Secretaria de Estado da Saúde de São Paulo, Araraquara, SP Brazil; 5 Setor de Pós-graduação, Pesquisa e Inovação, Faculdade de Medicina do ABC, Santo André, SP Brazil; 6 Fundação de Vigilância em Saúde do Amazonas, Manaus, AM Brazil; 7 Secretaria de Vigilância em Saúde, Departamento de Vigilância das Doenças Transmissíveis, Ministério da Saúde, Brasília, DF Brazil; 8 Faculdade de Medicina, Universidade de São Paulo, São Paulo, SP Brazil; 9 Departamento de Doenças Endêmicas Samuel Pessoa, Escola Nacional de Saúde Pública, Fundação Oswaldo Cruz, Rio de Janeiro, RJ Brazil; 10 Department of Disease Control, London School of Hygiene and Tropical Medicine, London, WC1E 7HT UK; 11 Escola de Matemática Aplicada, Fundação Getúlio Vargas, Rio de Janeiro, RJ Brazil

**Keywords:** Epidemiology, Transmission, Malaria metrics, Rural settlements, Brazilian Amazon

## Abstract

**Background:**

Brazilian malaria control programmes successfully reduced the incidence and mortality rates from 2005 to 2016. Since 2017, increased malaria has been reported across the Amazon. Few field studies focus on the primary malaria vector in high to moderate endemic areas, *Nyssorhynchus darlingi*, as the key entomological component of malaria risk, and on the metrics of *Plasmodium vivax* propagation in Amazonian rural communities.

**Methods:**

Human landing catch collections were carried out in 36 houses of 26 communities in five municipalities in the Brazilian states of Acre, Amazonas and Rondônia states, with API (> 30). In addition, data on the number of locally acquired symptomatic infections were employed in mathematical modelling analyses carried out to determine *Ny. darlingi* vector competence and vectorial capacity to *P. vivax*; and to calculate the basic reproduction number for *P. vivax*.

**Results:**

Entomological indices and malaria metrics ranged among localities: prevalence of *P. vivax* infection in *Ny. darlingi,* from 0.243% in Mâncio Lima, Acre to 3.96% in Machadinho D’Oeste, Rondônia; daily human-biting rate per person from 23 ± 1.18 in Cruzeiro do Sul, Acre, to 66 ± 2.41 in Lábrea, Amazonas; vector competence from 0.00456 in São Gabriel da Cachoeira, Amazonas to 0.04764 in Mâncio Lima, Acre; vectorial capacity from 0.0836 in Mâncio Lima, to 1.5 in Machadinho D’Oeste. The estimated *R*_0_ for *P. vivax* (*PvR*_0_) was 3.3 in Mâncio Lima, 7.0 in Lábrea, 16.8 in Cruzeiro do Sul, 55.5 in São Gabriel da Cachoeira, and 58.7 in Machadinho D’Oeste. Correlation between *P. vivax* prevalence in *Ny. darlingi* and vector competence was non-linear whereas association between prevalence of *P. vivax* in mosquitoes, vectorial capacity and *R*_0_ was linear and positive.

**Conclusions:**

In spite of low vector competence of *Ny. darlingi* to *P. vivax*, parasite propagation in the human population is enhanced by the high human-biting rate, and relatively high vectorial capacity. The high *PvR*_0_ values suggest hyperendemicity in Machadinho D’Oeste and São Gabriel da Cachoeira at levels similar to those found for *P. falciparum* in sub-Saharan Africa regions. Mass screening for parasite reservoirs, effective anti-malarial drugs and vector control interventions will be necessary to shrinking transmission in Amazonian rural communities, Brazil.

**Electronic supplementary material:**

The online version of this article (10.1186/s12936-019-2753-7) contains supplementary material, which is available to authorized users.

## Background

Both the morbidity and mortality associated with malaria have substantially decreased worldwide and in several endemic countries in Africa, South-East Asia and South America, including Brazil [1, 2]. However, in South-East Asian and South American countries, malaria control programmes face a challenging situation because of the high proportion of *Plasmodium vivax* malaria, lack of sustainable funding, and emerging resistance to anti-malarial drugs and available insecticides [1]. In Venezuela, South America, malaria increased by 365% from 2000 to 2015, with an additional 68% increase in 2017 [3]. In Brazil, malaria incidence increased by 47% in 2017 compared with 2015, including *Plasmodium falciparum* that emerged in areas previously free of this pathogen. The intensity of malaria transmission in Brazil is highly heterogeneous, with regions of high risk interspersed with others of moderate, low or no risk [4]. Furthermore, locations with the heaviest burden of malaria are those in the poorest and most remote regions of the Amazon River basin, in the states of Amazonas, Acre, Amapá, Pará and Roraima, with the occurrence of both *P*. *falciparum* and *P. vivax* (https://public.tableau.com/profile/mal.ria.brasil#!/), and the presence of subpatent or submicroscopic infection [5, 6]. Understanding spatiotemporal heterogeneities in the intensity and risk of transmission is important for planning and delineating measures and strategies to be adopted by an effective malaria control programme, as well as when disease incidence is decreasing, and elimination is forthcoming [7].

The dynamics of human *Plasmodium* transmission are shaped by complex interconnections of determinants, including those of vector biology, blood-feeding behaviour of vector species, temperature, precipitation, environment, ecology and human behaviour that determine the degree of human exposure to infectious mosquito bites [7–10]. Microclimatic changes caused by deforestation and forest degradation are associated with environmental and ecological changes that can increase the abundance of the primary mosquito vector *Ny. darlingi*, thereby increasing the malaria burden across Amazonian landscapes [11–13]. Comprehending the effects of environmental change on the malaria transmission cycle requires further knowledge of the locally relevant determinants, including entomological factors, such as the accurate identification of mosquito species competent for *Plasmodium* infection and directly involved in transmission [7]. The many components of malaria transmission include those of vector competence, vectorial capacity and transmission potential [14].

Mathematical models play an important role in investigating the mechanisms and processes that determine the transmission of malaria, and thus for malaria control and elimination. The mathematical models proposed by Ronald Ross and George Macdonald [8, 15] describe quantitatively the mechanisms involved in the dynamics of *Plasmodium* dispersion in a population composed of infected and susceptible hosts [9, 16]. The Ross–Macdonald model assumes homogeneous and random transmission of pathogens in a host population of indeterminate size [9]. The variables of the model include the population density of the mosquito vector in relation to humans, daily female survival, time required to complete a blood feeding cycle with subsequent oviposition, anthropophilism, and the extrinsic incubation period of the pathogen in the mosquito population. These parameters comprise the vectorial capacity that is the entomological variant of the basic reproductive rate for malaria (*R*_0_) [17]. Interventions for controlling malaria, such as the reduction of mosquito populations and human exposure to infectious bites, have been based largely on the components of the vectorial capacity formula of locally relevant mosquito species [16]. In addition to vectorial capacity, the basic reproduction number (*R*_0_) metric indicates the potential of an introduced *Plasmodium* species to propagate in a human community, given the local epidemiological data of malaria and ecological factors of the transmission cycle [7].

The Plan for Elimination of Malaria in Brazil proposed in 2015 by the Brazilian Ministry of Health includes the decline of both *P. vivax* and *P. falciparum* malaria across the Amazon River basin. However, in 2016–2017, Brazil was challenged by malaria resurgence, including *P. falciparum* malaria in areas where the pathogen had been eliminated. *Plasmodium vivax* malaria currently comprises more than 80% of the total malaria cases reported in Brazil [18]. A highly significant positive correlation between number of impacted forest patches less than 5 km^2^ and malaria cases were recently found, and that these patch sizes accounted for greater than ~ 95% of all patches in numerous study areas across the Brazilian Amazon for multiple years [13].

*Nyssorhynchus darlingi* is the primary malaria vector in several regions of the Brazilian Amazon River basin, and more broadly in the Amazon biome in South America. Despite its public health importance, field investigations focusing on the entomological components of malaria risk are few in number and restricted to a small portion of the massive geographical area of the Amazon. Moreover, vector competence and vectorial capacity of *Ny. darlingi* estimated employing contemporary entomological field data and locally acquired plasmodial infection in areas across the Brazilian Amazon are either very limited or nonexistent. Consequently, the need for sound and comprehensive field studies to empirically measure and evaluate the dimension and transmission potential of the most prevalent malaria in the Brazilian Amazon is necessary. The objectives of the study were to: (1) determine the vector competence and vectorial capacity of *Ny. darlingi* to *P. vivax* malaria; and (2) calculate the basic reproduction number (*R*_0_) for *P. vivax* (*PvR*_0_) malaria in five municipalities of the Brazilian Amazon. The primary focus of the analysis was *Ny. darlingi* because it was the most frequently found species in all localities, and *P. vivax* malaria because it comprised the great majority of cases during the period of field collections.

## Methods

### Study sites and mosquito adult collection

**Fig. 1:**
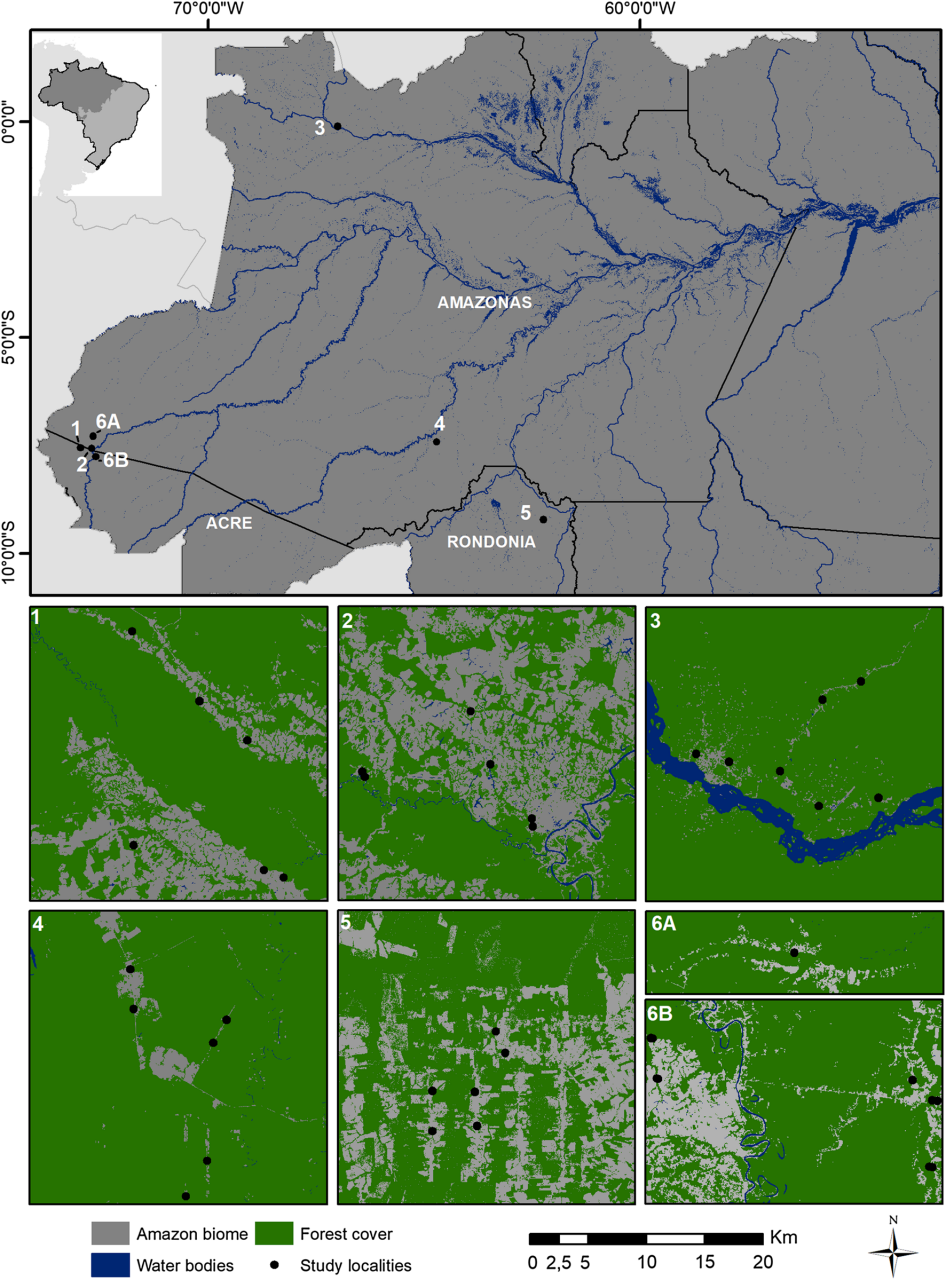
Specimen collection sites in five municipalities in the states of Acre, Amazonas and Rondônia, Brazil. Human landing catch (HLC) houses are represented by black dots. The numbers represent the municipalities as following: **1** Mâncio Lima; **2**, **6A**, **6B** Cruzeiro do Sul; **3** São Gabriel da Cachoeira; **4** Lábrea; **5** Machadinho D’Oeste

Females of the subfamily Anophelinae were collected in 36 houses of 26 communities in five municipalities in the Brazilian Amazon states of Acre, Amazonas and Rondônia states (Fig. 1; Table 1). The study locations were in rural settlements in areas with moderate to high endemicity of *P. vivax* malaria (Table 1; Additional file 1), where most infections were locally-acquired. To guide the choices of field collection communities, the mean annual malaria parasite incidence (annual parasite incidence, API ≥ 30) of local *P. vivax* infections in the previous several months and during the period of field collections in 2015 (http://portalms.saude.gov.br/saude-de-a-z/malaria/situacao-epidemiologica-dados), and 2017 (public data are available at https://public.tableau.com/profile/mal.ria.brasil#!/), as well as local information, were used as a proxy of the incidence. In addition, the selection of field localities was based on the level of forest cover, land use, and density of forest border as proxies of the presence of human and domestic animals. Coincidentally, the chosen areas were subject to discontinuous or negligible malaria control interventions, except for those measures focusing on either rapid or optical microscope diagnosis of *Plasmodium* infection in symptomatic humans, followed by immediate drug treatment. The use of insecticide-impregnated bed nets was either discontinuous or haphazardly employed in the communities.

**Table 1:** *Nyssorhynchus darlingi* collection data, including state, municipality, location, collection date (M/D/Y), collection time, local population, and *P. vivax* incidence, Brazil

State	Municipality	Location	Longitude	Latitude
Acre	Cruzeiro do Sul	Bairro Saboeiro	− 72.687972	− 7.637889
Acre	Cruzeiro do Sul	Bairro Cohab	− 72.688722	− 7.631889
Acre	Cruzeiro do Sul	Colonia Canela Fina	− 72.736139	− 7.549000
Acre	Cruzeiro do Sul	Colonia Igarapé Preto	− 72.720750	− 7.589972
Acre	Cruzeiro do Sul	Humaitá Ser	− 72.818000	− 7.599639
Acre	Cruzeiro do Sul	Humaitá Ser	− 72.820000	− 7.595639
Acre	Cruzeiro do Sul	Ramal Buritirana	− 72.709317	− 7.721300
Acre	Cruzeiro do Sul	Ramal Buritirana	− 72.714783	− 7.688950
Acre	Cruzeiro do Sul	Vila Lagoinha—Casa do Sr. João	− 72.486083	− 7.738667
Acre	Cruzeiro do Sul	PDS Jamil Jereissati—Ramal Zacarias	− 72.665670	− 7.285750
Acre	Cruzeiro do Sul	PDS Jamil Jereissati—Ramal do Caracas	− 72.490350	− 7.792033
Acre	Mâncio Lima	Bairro Guarani	− 72.885559	− 7.620124
Acre	Mâncio Lima	Bairro Guarani	− 72.870281	− 7.625893
Acre	Mâncio Lima	Colonia Normando	− 72.985791	− 7.600644
Acre	Mâncio Lima	Colonia Paraná do Pentecoste	− 72.898319	− 7.520125
Acre	Mâncio Lima	Colonia Paraná do Pentecoste	− 72.935207	− 7.489624
Acre	Mâncio Lima	Colonia Paraná do Pentecoste	− 72.987000	− 7.436000
Amazonas	Lábrea	PA Umari, Boa Água, Road BR-230 km 24	− 64.676783	− 7.404668
Amazonas	Lábrea	PA Umari, Boa Água, Road BR-230 km 24	− 64.666417	− 7.386668
Amazonas	Lábrea	PA Paciá, Road BR-230 km 26	− 64.697738	− 7.522993
Amazonas	Lábrea	PA Paciá, Road BR-230 km 26	− 64.681215	− 7.495510
Amazonas	Lábrea	PA Umari, Apairal, Road BR-230	− 64.740907	− 7.347613
Amazonas	Lábrea	PA Umari, Apairal, Road BR-230	− 64.738264	− 7.378220
Rondônia	Machadinho D’Oeste	Belo Horizonte, Galo Velho, LH TB 14	− 62.237460	− 9.177300
Rondônia	Machadinho D’Oeste	Belo Horizonte, Galo Velho, LH TB 13	− 62.230107	− 9.193528
Rondônia	Machadinho D’Oeste	Belo Horizonte, Galo Velho, LH 10	− 62.253619	− 9.223432
Rondônia	Machadinho D’Oeste	Belo Horizonte, Galo Velho, LH 10	− 62.251806	− 9.249693
Rondônia	Machadinho D’Oeste	Belo Horizonte, Galo Velho, LH 9	− 62.286519	− 9.253933
Rondônia	Machadinho D’Oeste	Belo Horizonte, Galo Velho, LH 9	− 62.286399	− 9.222947
Amazonas	São Gabriel da Cachoeira	Road BR-307 km 15	− 67.001667	− 0.071389
Amazonas	São Gabriel da Cachoeira	Road Camanaus, Itacoatiara Mirim	− 67.004722	− 0.153611
Amazonas	São Gabriel da Cachoeira	Road Porto Camanaus	− 66.958611	− 0.147222
Amazonas	São Gabriel da Cachoeira	District Tiago Montalvo	− 67.099167	− 0.113056
Amazonas	São Gabriel da Cachoeira	District Miguel Quirino	− 67.073889	− 0.119167
Amazonas	São Gabriel da Cachoeira	Boa Esperança, Road BR-210 km 7	− 67.034167	− 0.126667
Amazonas	São Gabriel da Cachoeira	Road BR-307 km 19	− 66.971944	− 0.057222

Coll date	Coll time	Local population	Local *P. vivax* malaria incidence^a^	
Apr 19, 2015	18 h:00–0 h:00	2239	53.6	
Apr 20, 2015	18 h:00–0 h:00	1258	42.9	
Apr 21, 2015	18 h:00–0 h:00	426	86.9	
Apr 24, 2015	18 h:00–0 h:00	634	71.0	
Apr 23, 2015	18 h:00–0 h:00	127	236.2	
Apr 25, 2015	18 h:00–0 h:00	127	236.2	
Jul 01, 2017	18 h:00–0 h:00	548	104	
Jul 07, 2017	18 h:00–0 h:00	548	104	
Jul 21, 2017	18 h:00–0 h:00	557	222.6	
Jul 22, 2017	18 h:00–0 h:00	110	381.8	
Jul 23, 2017	18 h:00–0 h:00	271	284.1	
May 28, 2015	18 h:00–0 h:00	1174	36.6	
May 29, 2015	18 h:00–0 h:00	1174	36.6	
May 30, 2015	18 h:00–0 h:00	73	82.2	
Jun 01, 2015	18 h:00–0 h:00	544	240.8	
Jun 03, 2015	18 h:00–0 h:00	544	240.8	
Jun 04, 2015	18 h:00–0 h:00	544	240.8	
Jul 31, 2015	18 h:00–0 h:00	163	165.6	
Aug 02, 2015	18 h:00–0 h:00	163	165.6	
Aug 04, 2015	18 h:00–0 h:00	286	83.9	
Aug 05, 2045	18 h:00–0 h:00	286	83.9	
Aug 09, 2015	18 h:00–0 h:00	68	264.7	
Aug 10, 2015	18 h:00–0 h:00	68	264.7	
Oct 19, 2015	18 h:00–0 h:00	129	38.8	
Oct 21, 2015	18 h:00–0 h:00	111	81.1	
Oct 22, 2015	18 h:00–0 h:00	180	72.2	
Oct 23, 2015	18 h:00–0 h:00	180	72.2	
Oct 26, 2015	18 h:00–0 h:00	90	144.4	
Oct 27, 2017	18 h:00–0 h:00	90	144.4	
Nov 09, 2017	18 h:00–0 h:00	73	835.6	
Nov 11, 2017	18 h:00–0 h:00	259	173.7	
Nov 18, 2017	18 h:00–0 h:00	164	115.9	
Nov 14, 2017	18 h:00–0 h:00	1081	368.2	
Nov 15, 2017	18 h:00–0 h:00	2128	149.9	
Nov 21, 2017	18 h:00–0 h:00	60	200	
Nov 17, 2017	18 h:00–0 h:00	4	3250	

Geodetic Datum WGS84

^a^*P. vivax* malaria incidence in the previous and/or in the month of human landing catch field-collections in the rural locations studied

The municipalities of Cruzeiro do Sul and Mâncio Lima are in the region of the Juruá River basin, western Acre state; Machadinho D’Oeste is situated in the Machadinho River basin, along highway BR-364, and Lábrea municipality is alongside the Boa Água River, a tributary of the Purus River, west of the BR-230 Brazilian Trans-Amazonian highway. The most northern municipality, São Gabriel da Cachoeira, is in northwestern Brazil at the frontier of Colombia and Venezuela, in the Upper Negro River region (Fig. 1).

The municipalities of Cruzeiro do Sul, Mâncio Lima and Lábrea have a tropical climate with significant rainfall most of the year, and a short dry season. The climate is classified as Am according to Köppen and Geiger with average temperature varying from 26.4 °C in Lábrea to 25.3 °C in Cruzeiro do Sul, and average annual rainfall values of 2139 mm (Cruzeiro do Sul) to 2318 mm (Lábrea). Machadinho D’Oeste has a tropical climate, with less rainfall in the winter than in the summer. According to Köppen and Geiger, the climate classification is Aw, with an average temperature of 25.3 °C, and average annual rainfall of 2117 mm. The climate of São Gabriel da Cachoeira region is tropical with significant rainfall throughout the year, even during the driest month. According to Köppen and Geiger, this climate classification is Af, with the average annual temperature of 26.4 °C, and the average annual rainfall of 2909 mm (https://pt.climate-data.org/america-do-sul/brasil/).

Female adult collections were conducted from April to November, during the wet–dry transition, and in the dry season (Table 1). Collections were outdoors in the peridomestic environment within ~ 5 m of each of 36 houses. Considering the 26 rural communities separately, houses chosen for human landing catch (HLC) were at least 2.5 km apart and were positioned in the centre of a 1 km radius circle to avoid sampling more than one house within the same 3.14 km^2^ area. Each black dot in Fig. 1 represents a house that was selected for conducting the HLC mosquito collections. Although there was active malaria transmission and reported cases in all communities (Table 1), house selection within rural communities was not based on malaria cases among the residents in the collection period of time. Furthermore, selection was also guided by the level of forest cover, land use, and density of forest border (Fig. 1).

Human landing catch collections were carried out one night each 36 houses, from 18 h:00 until 0 h:00 (Table 1). Following the field protocol, it was possible to collect in more than one house in some locations, for example, in PA Umari, Boa Água (Sivep location 163), two houses were sampled, each collection was carried out one night from 18 h:00 until 0 h:00. One or two collectors worked each night. Variation in the number of collectors was due to their availability during the field trip. The mosquito sampling effort was 216 h-collection, distributed as follows: Lábrea, Machadinho D’Oeste and Mâncio Lima 36 h each, São Gabriel da Cachoeira 42 h, and Cruzeiro do Sul 66 h. Every hour, female mosquitoes were euthanized with ethyl acetate (C_4_H_8_O_2_) vapors in the field and stored in silica gel separated by date, location, house and hour of collection. Specimens were morphologically identified to species level by MAMS, labeled and stored individually with silica gel at room temperature for subsequent analysis.

### Mosquito processing

Genomic DNA was extracted from whole, adult female *Ny. darlingi* using Qiagen DNeasy Blood & Tissue Kit (Hilden, Germany). All *Ny. darlingi* DNA samples were tested for *Plasmodium* spp. infection following [19], with DNA pools of up to five individuals containing equal amounts of gDNA. Mosquito samples with DNA concentrations of < 1.0 ng/µL or > 15 ng/µL were tested individually and not pooled. In instances where the species of *Plasmodium* could not be detected with the triplex assay, PCR amplification and agarose gel (2%) electrophoresis of PCR products was performed using primer pairs for *P. vivax* and *P. falciparum* [20]. Each PCR reaction contained 1× PerfeCTa qPCR ToughMix, Uracil *N*-glycosylase (UNG), ROX (Quanta Biosciences, USA), 0.3 μM of each primer, ultrapure water, and 2 μL genomic DNA, with a total volume of 20 µL. Cycling conditions were as follows: 5 min UNG-activation hold at 45 °C and a denaturation step for 10 min at 95 °C, followed by 50 cycles of 95 °C denaturation for 15 s and 60 °C annealing/elongation for 1 min.

### Malaria epidemiological data

Data on the number of cases of malaria by epidemiological week and annual parasite incidence (IPA) of *P. vivax* (Table 1; Additional file 1) were requested by first author from the Ministry of Health, Sistemas de Informações de Vigilância Epidemiológica (SIVEP) Malaria [18], through the Electronic System of the Citizen Information Service (Sistema Eletrônico do Serviço de Informações ao Cidadão - e-SIC) (https://esic.cgu.gov.br/sistema/site/index.aspx), protocols # 25820001316201742, # 25820003892201813, # 25820004426201847, and # 25820004717201835. Because asymptomatic infections are not included in the SIVEP Malaria [18] platform, only locally acquired symptomatic infections were employed in the mathematical modelling analysis.

### Human-biting rate, prevalence of infection in mosquito, estimation of vector competence, vectorial capacity and basic reproduction number

Vector competence is defined as “the ability of a vector to transmit a disease” [21]. It normally comprises the capacity of a vector to be infected, maintain and transmit an infectious agent. In the present study, vector competence was defined as the probability that an infected mosquito generates a new infection when it bites a susceptible human [14]. Vector competence was initially defined by [22] in pioneering work on malaria modelling. Vector competence is denoted *b* throughout the current paper.

The analysis began by considering malaria incidence in the studied rural communities (Table 1). The data were obtained from SIVEP Malaria [18] through the Citizen Information Service, Ministry of Health of Brazil (https://esic.cgu.gov.br/sistema/site/index.aspx). The incidence of malaria was calculated in rural communities at the time of mosquito collection, denoted *Incidence(t)*, that is the number of new malaria cases per unit time. It is equal to the product of the force of infection, *λ* (incidence-density rate or *per capita* incidence), times the number of susceptible humans, *S*_*H*_(*t*). It is also the product of the ratio of mosquitoes-to-humans, *N*_*M*_(*t*)/*N*_*H*_(*t*), times the mosquito biting rate, *a*, times the vector competence, *b* (see below), times the prevalence of *Plasmodium* infection in the mosquitoes, $I_{M} (t)/N_{M} (t)$, times the number of susceptible humans, $S_{H} (t)$, or:

1 $$Incidence\left( t \right) = \lambda \left( t \right)S_{H} \left( t \right) = \frac{{N_{M} \left( t \right)}}{{N_{H} }}ab\frac{{I_{M} \left( t \right)}}{{N_{M} \left( t \right)}}S_{H} \left( t \right)$$

Incidence data from the five municipalities studied were then fitted to the continuous function:

2 $$Incidence\left( t \right) = \alpha \exp (\beta t)$$

**Fig. 2:**
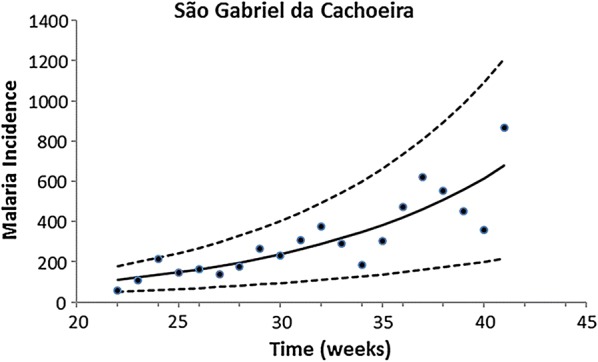
Fitting Eq. (2) to the data from São Gabriel da Cachoeira. Dots represent real data from SIVEP Malaria [18], the continuous line the mean, and the dotted lines the 95% confidence interval (C.I.). The fitting parameters are α = 14 (95% C.I. 10–20) and β = 0.095 (95% C.I. 0.075–0.100)

**Fig. 3:**
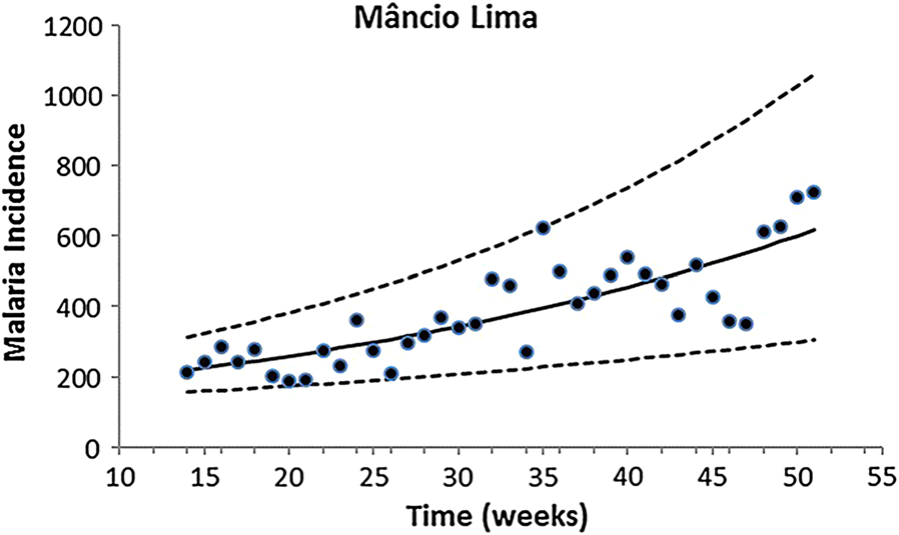
Fitting Eq. (2) to the data from Mâncio Lima. Dots represent real data from SIVEP Malaria [18], the continuous line the mean, and the dotted lines the 95% confidence interval (C.I.). The fitting parameters are α = 148.25 (95% C.I. 121.26–197.30) and β = 0.028 (95% C.I. 0.018–0.033)

**Fig. 4:**
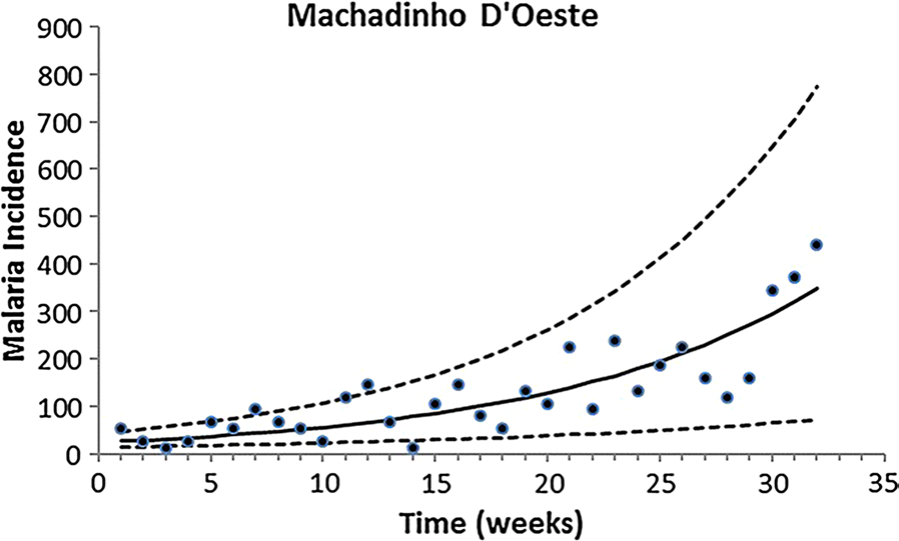
Fitting Eq. (2) to the data from Machadinho D’Oeste. Dots represent real data from SIVEP Malaria [18], continuous line show the mean the dotted lines with 95% confidence interval (C.I.). The fitting parameters are α = 24.45 (95% C.I. 13.21–43.21) and β = 0.083 (95% C.I. 0.053–0.900)

**Fig. 5:**
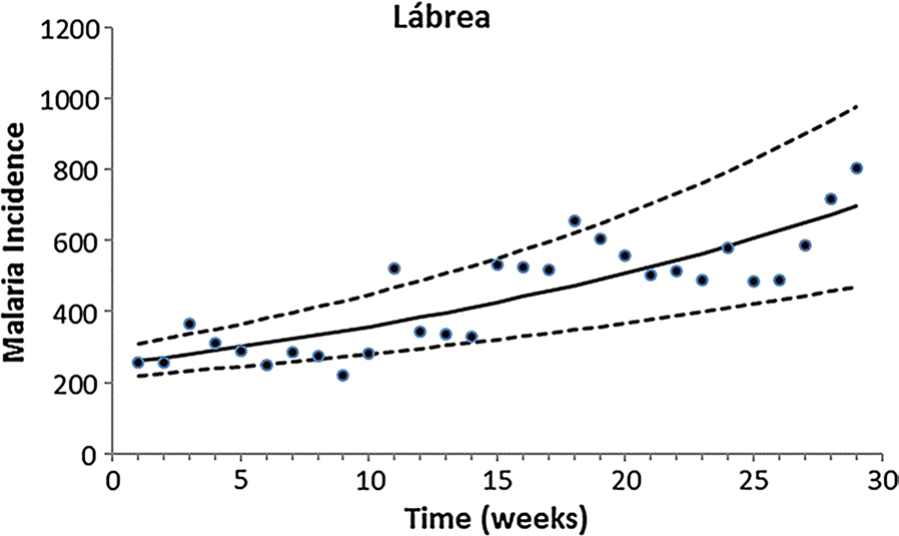
Fitting Eq. (2) to the data from Lábrea. Dots represent real data from SIVEP Malaria [18], the continuous line the mean, and the dotted lines the 95% confidence interval (C.I.). The fitting parameters are α = 252.32 (95% C.I. 214.22–297.61) and β = 0.035 (95% C.I. 0.027–0.041)

**Fig. 6:**
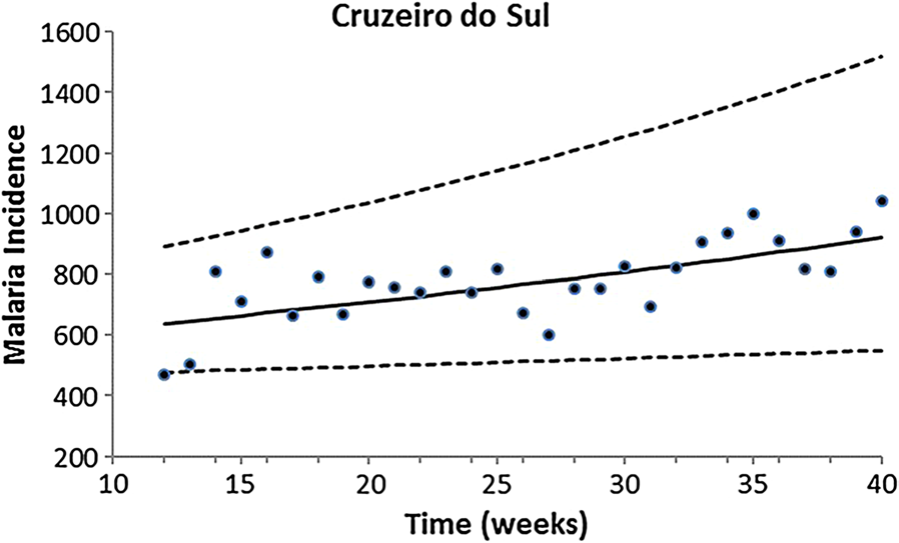
Fitting Eq. (2) to the data from Cruzeiro do Sul. Dots represent real data from SIVEP Malaria [18], the continuous line the mean, and the dotted lines the 95% confidence interval (C.I.). The fitting parameters are α = 546.59 (95% C.I. 449.85–709.04) and β = 0.013 (95% C.I. 0.005–0.019)

Fitting of the incidence data was restricted to the period of data collection, which occurred in the same season for all localities. Therefore, only the growing phase of the annual malaria seasonal cycle was considered for this model. The exponential form chosen was restricted to the period of the study. Figures 2, 3, 4, 5 and 6 show the fitting of Eq. (2) to the actual data on malaria incidence in each of the five collection localities.

As mentioned above, vector competence, *b*, is the probability that a new human infection is produced when a fraction of infected mosquitoes bite humans with rate *a*. It can be calculated from the incidence of malaria, using the equation:

3 $$b_{t} = \frac{{Incidence_{t} }}{{aS_{Ht} }}\frac{{N_{H} }}{{N_{Mt} }}\frac{{N_{Mt} }}{{I_{Mt} }}$$

where *t* is the moment of data collection. Therefore, given at day *t*: the malaria incidence at the site and the malaria prevalence in the mosquitoes, calculating the human-biting rate (HBR) from the HLCs (see below) and the number of susceptible humans from the history of malaria, the vector competence at day *t* from Eq. (3) can be calculated.

As mentioned above, the HBR is the number of bites that a mosquito population inflicts on humans per unit time [23]. In this study, the HBR was obtained by the number of mosquitoes collected on HLC per night per catcher. It is the product of the ratio of mosquitoes-to-humans, $N_{M} (t)/N_{H} N_{M} (t)/N_{H}$, times the mosquito daily biting rate, *a*:

4 $$HBR_{t} = a\frac{{N_{Mt} }}{{N_{H} }}$$

HBR_t_ values for the five municipalities studied were obtained from the number of mosquitoes captured by HLC per collector, per night, at day *t*. It is assumed that captured mosquitoes approached the collector to bite. Therefore, the number of mosquitoes collected in each HLC corresponds to the HBR_t_ of these specific mosquitoes in these specific collection sites.

To estimate the probability of acquiring malaria at day *t*, that is, on the day of the field collections, the weekly incidence data, available from SIVEP Malaria [18], were disaggregated. For this the following procedures were applied. Firstly, it was calculated the number of malaria cases in the time interval between *t*_0_ and *t*_1_, which is simply the integral of the incidence in this interval, calculated by the equation:

5 $$Cases = \int\limits_{{t_{0} }}^{{t_{1} }} {\lambda (t)S_{H} (t)} dt = \int\limits_{{t_{0} }}^{{t_{1} }} {ab\frac{{I_{M} (t)}}{{N_{H} }}S_{H} (t)dt}$$

The daily number of malaria cases between week *t*_0_ and week *t*_1+1/7_, *H*_*t*_, therefore, is the difference between the number of malaria cases in the time interval between *t*_0_ and *t*_1+1/7_ minus the number of malaria cases in the time interval between *t*_0_ and *t*_1_. It is calculated by the equation:

6 $$H_{t} = \mathop \int \limits_{{t_{0} }}^{{t_{1 + 1/7} }} \lambda \left( t \right)S_{H} \left( t \right)dt - \mathop \int \limits_{{t_{0} }}^{{t_{1} }} \lambda \left( t \right)S_{H} \left( t \right)dt$$

In the calculation of the daily incidence, it was assumed a stable situation, and estimated the remaining susceptible population, *S*_*H*_, from the annual parasite incidence (API), and subtracted the number of parasite positive individuals from the total population of each of the studied locations.

The vectorial capacity at day *t* (*V*_*Ct*_) [24] is the daily number of potential infections produced by a single infective individual, *I*_*H*_. It is calculated from the per capita daily incidence, $h_{t} = \frac{{H_{t} }}{{S_{Ht} }}$, by the equation [25, 26]:

7 $$h_{t} = b_{t} \left[ {1 - \exp \left( { - V_{C} I_{H} (t - \tau )t} \right)} \right]\quad \Rightarrow V_{Ct} = \frac{{ - \ln \left( {1 - \frac{{h_{t} }}{{b_{t} }}} \right)}}{{I_{H} (t - \tau )}}$$

where *τ* is the total incubation period (both extrinsic inside the mosquito and intrinsic inside the human hosts) and was assumed to be equal to 1 week, and *b*_*t*_ is now:

8 $$b_{t} = \frac{{h_{t} }}{{aS_{Ht} }}\frac{{N_{H} }}{{N_{Mt} }}\frac{{N_{Mt} }}{{I_{Mt} }} = \frac{{h_{t} }}{{HBR_{t} S_{Ht} }}\frac{{N_{Mt} }}{{I_{Mt} }}$$

The first part of Eq. (7) is the same as the one originally presented in [25] with different notation. It can be interpreted as follows. Assuming a Poisson distribution, the probability of receiving no potentially infective inoculation is $\exp \left( { - V_{C} I_{H} (t - \tau )t} \right)$, and the probability of receiving at least one potentially infective inoculation is $1 - \exp \left( { - V_{C} I_{H} (t - \tau )t} \right)$, a fraction *b*_*t*_ of which is actually infective. Note that *h* is interpreted in Eq. (7) as the probability of one malaria case produced by one single infective human individual *I*_*H*_ who acquired the infection *t *− *τ* days ago. Note that the “difference” *t *− *τ* is not computed in the calculations.

The basic reproduction number, *R*_0_, is the number of infections produced by a single infective individual, *I*_*H*_, in an entirely susceptible population throughout his/her infectiousness period, 1/*γ* [27]. It is calculated from the vectorial capacity by the equation:

9 $$R_{0} = V_{Ct} \frac{c}{\gamma }$$

where *c* is the probability that an infected individual when bitten by a susceptible mosquito generates a new infection in the mosquito [22]. Although the value of *R*_0_ varies throughout the seasons of the year, this value was calculated for the day of data collection.

## Results

The human population in each municipality, and results of the variables from the field-collected data employed in the mathematical modelling, namely, the *P. vivax* annual parasite incidence (API), the remaining susceptible human population (S_H_), the prevalence of *P. vivax* infection in *Ny. darlingi* mosquitoes, $I_{Mt} /N_{Mt}$, and the daily human-biting rate per human collector, $HBR_{t}$, are shown in Table 2 and Additional file 2.

**Table 2:** Geographical locality, human population, annual parasite index (API) of *P. vivax*, remaining susceptible population (S_*H*_), prevalence of *P. vivax* in *Ny*. *darlingi*, and daily human biting rate per person (HBR) calculated using data of 216 h human landing catch (HLC) in the peridomestic environment of 36 houses in rural settlements of five municipalities in the Brazilian states of Acre, Amazonas and Rondônia

Municipality (State)^a^	Human population	API^b^ *P. vivax*	S_*H*_^c^	Prevalence of *P. vivax* infection in mosquito (%)^d^	HBR_*t*_^e^
Mâncio Lima (AC)	18,708	241	11,852	0.243	33 ± 1.50
Lábrea (AM)	40,969	279	29,530	0.246	66 ± 2.41
Cruzeiro do Sul (AC)	80,168	236	67,400	1.5	23 ± 1.18
São Gabriel da Cachoeira (AM)	48,760	150	41,410	2.9	52 ± 4.83
Machadinho D’Oeste (RO)	19,367	144	16,631	3.96	39 ± 1.21

^a^AC (Acre state), AM (Amazonas state), RO (Rondônia state)

^b^Annual parasite index = proportion of individuals with circulating malaria parasites

^c^S_*H*_ = remaining susceptible human population [for details see “Methods” and Eq. (6)]

^d^Obtained by RT-PCR from single field collected mosquitoes

^e^Human biting rate, the per capita number of bites per night, calculated with the data obtained from the HLC based on 6-h collection

Results of the analyses in Table 2 demonstrated that all five studied locations present high levels of *P. vivax* malaria endemicity. This is reflected mainly in the high API values that range from 144 in Machadinho D’Oeste to 279 in Lábrea, and the high prevalence of *Ny. darlingi* to *P. vivax* infection. In Lábrea, the estimated HBR was very high, reaching 66 bites per human per 6-h per night, with 0.246% of *Ny. darlingi* found RT-PCR positive for *P. vivax*, and the API value was 279 per 1000 population. The highest values of prevalence of *P. vivax* infection in *Ny. darlingi* were found in Machadinho D’Oeste (3.96%), São Gabriel da Cachoeira (2.9%) and Cruzeiro do Sul (1.5%). The estimated HBRs were 23 in Cruzeiro do Sul, 39 in Machadinho D’Oeste, 52 in São Gabriel da Cachoeira and 66 in Lábrea. The API ranged from 144 in Machadinho D’Oeste, 150 in São Gabriel da Cachoeira and 279 in Lábrea. The second highest API value (API = 241) was estimated for Mâncio Lima, and the prevalence of *P. vivax* infection in *Ny. darlingi* was 0.243%, a value slight lower than that found in Cruzeiro do Sul.

Results of mathematical modelling analysis to estimate entomological parameters of malaria, namely, the daily malaria incidence, *h*_*t*_; vector competence, *b*_*t*_; vectorial capacity, $V_{Ct}$; and the basic reproduction number, *R*_0_ are shown in Table 3.

**Table 3:** Results of the estimated parameters (mean and 95% confidence interval) for *P. vivax*, using human landing catch conducted outdoors in the peridomestic environment of 36 houses in rural settlements of five municipalities in the Brazilian states of Acre, Amazonas and Rondônia

Municipality (State)^a^	Estimated malaria incidence at day *t* (*h*_*t*_)	Malaria probability at day *t*	Vector competence (*b*_*t*_)	Vectorial capacity (days^−1^) (*Vc*_*t*_)	Basic reproduction number^b^ (*R*_0_)
Mâncio Lima (AC)	45 (28–69)	0.0038 (0.0024–0.0058)	0.0476 (0.0306–0.0703)	0.0836 (0.0809–0.0862)	3.3 (3.2–3.4)
Lábrea (AM)	100 (67–140)	0.0034 (0.0023–0.0047)	0.0208 (0.0144–0.0283)	0.177 (0.171–0.183)	7.0 (6.8–7.2)
Cruzeiro do Sul (AC)	100 (74–173)	0.00167 (0.00110–0.00256)	0.00484 (0.00332–0.00702)	0.42 (0.40–0.45)	16.8 (15.9–17.7)
São Gabriel da Cachoeira (AM)	142 (42–259)	0.00344 (0.00101–0.00626)	0.00456 (0.00137–0.00814)	1.4 (1.3–1.5)	55.5 (53.3–57.9)
Machadinho D’Oeste (RO)	174 (23–429)	0.0104 (0.0014–0.0258)	0.0135 (0.0018–0.035)	1.5 (1.4–1.6)	58.7 (55.4–62.3)

^a^AC (Acre state), AM (Amazonas state), RO (Rondônia state)

^b^Obtained from Eq. (8) with *c *= 0.22 [28], and *γ* = 5.56 × 10^−3^ days^−1^ [29]

The vector competence (*b*_*t*_) values ranged tenfold from the lowest in São Gabriel da Cachoeira (0.00456) to the highest in Mâncio Lima (0.0476). These differences were inversely correlated with the malaria intensity metrics, the vectorial capacity (*V*_*Ct*_) and the basic reproduction number (*R*_0_). The *V*_*Ct*_ calculated for HLC *Ny. darlingi* in São Gabriel da Cachoeira was 1.4 (1.3–1.5), whereas in Mâncio Lima it was 0.0836 (0.0809–0.0862). This variation was likely due to the indirect way vector competence was estimated. For the observed high malaria incidence and prevalence of infection in the vector, very low vector competence suffices to explain the data. The two highest estimated malaria incidences at day *t* values were found in Machadinho D’Oeste (174) and São Gabriel da Cachoeira (142), and the lowest value in Mâncio Lima (45). The highest probability of having malaria at day *t* was found in Machadinho D’Oeste (0.0104), whereas the lowest was in Cruzeiro do Sul (0.00167).

**Fig. 7:**
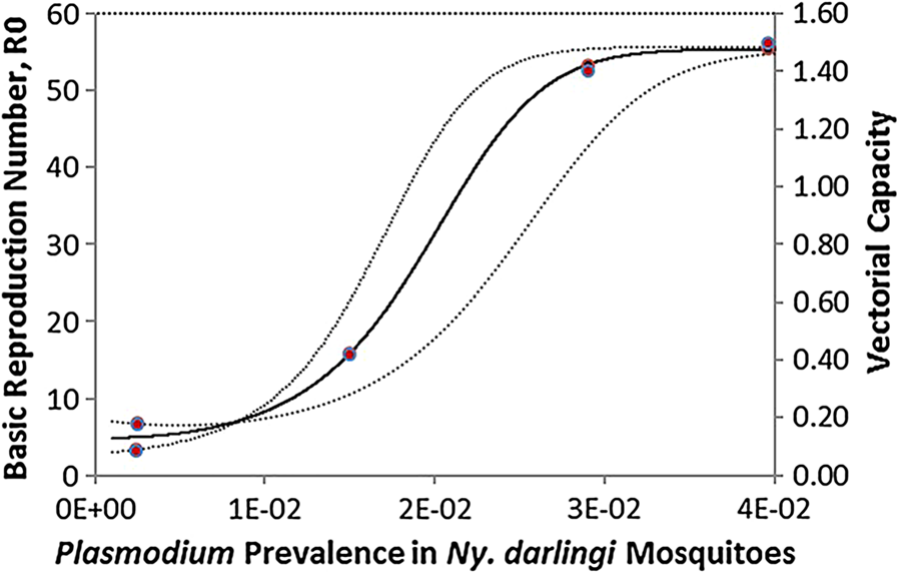
Correlation between the *P. vivax* prevalence in the mosquitoes and both the vectorial capacity (red dots) and the basic reproduction number (black dots) (*R*^2^= 0.998)

Figure 7 shows the correlation between the prevalence of *P. vivax* in the mosquitoes and both the vectorial capacity and the basic reproduction number (*R*_0_). The association is clearly linear, and the trend is positive. The difference between the vectorial capacity and the basic reproduction number is only by scale since the latter is the result of the multiplication of the former by a constant $\frac{c}{\gamma }$. The association between both the vectorial capacity and the basic reproduction number and the prevalence of *P. vivax* in the mosquitoes is highly non-linear. This non-linearity can be understood if Eq. (8) substitutes into Eq. (7) to obtain:

10 $$V_{Ct} = \frac{{ - \ln \left[ {1 - \left( {\frac{{I_{Mt} }}{{N_{Mt} }}\frac{1}{{HBR_{t} S_{Ht} }}} \right)} \right]}}{{I_{H} (t - \tau )}}$$

## Discussion

Strategic planning for malaria control and prevention are usually tailored based on categorization of API, without knowledge of the determinants of malaria risk that impact the propagation of *Plasmodium* parasite and intensify transmission in a region [7]. Thus, the success in reducing the disease incidence is continuously threatened by a combination of multiple determinants that can increase intensity of malaria transmission. For example, anthropogenic changes in the forest environment that increase the local mosquito vector potential [12, 13], in addition to human-related determinants [7]. Among a multitude of factors, intensive human movement can support the introduction of new infections acquired in other areas with active transmission. In addition, poor local housing conditions, economic failures, insufficient financial investment in malaria control and poorly managed surveillance programmes can favour the reemergence of malaria and intensify transmission to higher levels [7, 10].

The choice of rural settlements to conduct mosquito collections was not random. Localities and municipalities with active malaria transmission and reported cases in the previous and/or in the month of field collections were selected (Table 1). This approach somewhat biased the data because it tends to overestimate the number of infected mosquitoes, especially in areas where transmission is expected to be heterogeneous. To minimize the bias, the selection of houses for HLC collections was also guided by the level of forest cover, land use, and density of the forest border (Fig. 1). In addition, houses were at least 2.5 km apart to avoid sampling in houses situated within a circle of 1-km radius. What is more, considering that this is a methodological study conducted to estimate the malaria metrics in rural communities with moderate to high transmission intensity, the choice for field-collections does not invalidate the results of the analyses. Furthermore, this is the first study to calculate the vectorial capacity of vectors based on empirical data of malaria incidence in Brazil.

In the analyses, it was used empirical data from mosquito field-collections and reported data on local malaria incidence to calculate, through simple mathematical techniques, three key parameters related to malaria transmission intensity: vector competence, vectorial capacity and basic reproduction number (*R*_0_). The mathematical approach adopted in this study consists of equations relating the reported malaria incidence to vector competence and vectorial capacity. From the latter, it was calculated *PvR*_0_ in the Amazonian rural communities. Likely, the mathematical approach has limitations, for instance, it is assumed that all individuals in a certain population are identical and equally exposed to *Plasmodium* infection. It is known that human-to-mosquito transmission efficiency may change as malaria prevalence changes, however it was assumed a fixed value for all localities, obtained from the published literature. This is due to the lack of studies addressing this important parameter. As the major objective is to propose a new method for calculating the malaria metrics, this limitation does not invalidate the approach adopted in the study. However, further studies on this topic will be necessary to improve the methodological approach proposed. Another limitation is subpatent or submicroscopic infections that were not considered in the analysis because of lack of information for the localities studied. Inclusion of asymptomatic infections in the analyses could impact the scale, but the method would be the same.

The proportion of *Ny. darlingi* in HLC collections in the peridomestic environment was similar to values obtained in previous studies carried out in other areas across the Brazilian Amazon [30–33]. Similarities among results of diverse studies may be caused by the high degree of anthropophily of *Ny. darlingi*. This mosquito vector is specially attracted to human-specific volatiles, hence the effectiveness of HLC compared with CDC-LT and the mosquito magnet trap, among other trap types [34–37]. In the current study, the number of *Ny. darlingi* mosquitoes on HLC collectors ranged from 23 to 66 specimens per collector per 6 h night (Table 1). These findings are of the same order of magnitude as those by Tadei and collaborators [30] in the Brazilian Amazon. However, the range is narrower compared with a recent study from Amazonian Peru, where using HLC in the peridomestic environment, the number of *Ny. darlingi* in the dry season versus the rainy season per collector per 12 h night ranged from 1.5 to 250.5 specimens (average of two nights) [38]. In the heterogeneous Amazonian landscapes, spatial distribution and prevalence of *Ny*. *darlingi* depends on the level of precipitation, temperature, presence of standing water, level and pattern of forest fragmentation, size and abundance of deforested patches, proximity of larval habitats to human houses, and reallocation of water associated with land-use [39–43]. Thus, the range from 23 to 66 mosquitoes per catcher per 6 h night is likely due to a combination of environmental and ecological factors that influence the seasonal distribution of the species. In addition, distinct from Iquitos, field collections in the localities studied were carried out in the wet-dry and dry seasons, when malaria transmission intensifies in the Brazilian Amazon.

The results of the mathematical modelling conducted to estimate the prevalence of *P. vivax* in *Ny. darlingi* are similar to the levels of variation found in previous studies. Typically, *Ny. darlingi* vector infection rate has been recorded in the published literature as ~ 1–2% [37, 44, 45]. In contrast, in some high-malaria risk areas in the Brazilian Amazon, the prevalence rate of *Plasmodium* in *Ny. darlingi* was unexpectedly low (less than 0.5%) [30, 46]. This could be explained by the relatively low number of specimens examined (n = 308), even though it was during the peak malaria transmission season (July–August). Local variation in the prevalence rate was also detected in districts of the municipality of Boa Vista, Roraima. Whereas Silva Vasconcelos et al. [47] found an 8.5% *Plasmodium* prevalence rate in *Ny. darlingi* from two riverine districts, Póvoa et al. [48] showed that the overall *Plasmodium* infection rate was 2.1% in six distinct districts in the same municipality. The highest prevalence of *P. vivax* infection in *Ny. darlingi* (approximately 4%) was found in Galo Velho settlement (Table 2) in Machadinho D’Oeste, Rondônia. This value is closer to the infection rate found in *Anopheles funestus* in Tanzania (6.3% according to Taylor [49]) than to that of a Neotropical malaria vector. The second highest prevalence of *P. vivax* infection in the main vector (approximately 2.9%) was detected in São Gabriel da Cachoeira, Amazonas. Although the infection rate was lower than in Machadinho D’Oeste, the HBR was ~ 52 in São Gabriel da Cachoeira, whereas in Machadinho D’Oeste it was ~ 39. Considering the entomological potential for malaria transmission, mathematically quantified by the vectorial capacity (Table 3), one can assume that the effectiveness of *Ny. darlingi* in propagating *P. vivax* throughout in local population is similar. Interventions for reducing the number of mosquito infectious bites per human per day, and the overall number of mosquito bites, should be prioritized by malaria control programmes. Malaria commodities for vector control such as insecticide impregnated nets, indoor insecticide spraying, management of larval habitats, environmental modifications and household improvements can be appropriate for reducing human exposure to vectors. However, effectiveness of the vector control measures will depend on knowledge of mosquito vector biology and the environment that sustain the population [7].

Vector competence is defined in this study as the probability of one infected *Ny. darlingi* mosquito generating a human case of *P. vivax* malaria when biting a non-infected person. Results of the analyses showed relatively low values (from 0.46 to 4.8%). The tenfold variation observed is more likely due to local environmental characteristics rather than biological variation in *P. vivax* and *Ny. darlingi* from the various collecting locations. However, the potential for biological variation between sub-populations of *Ny. darlingi* with distinctive competencies for *P. vivax* cannot be excluded. By fitting their models to field data on malaria prevalence, Dietz et al. [25, 26] estimated that vector competence (*g* in their notation) resulted in 9.7% (± 1.7%) for *Anopheles gambiae* and *An*. *funestus* in Nigeria. This high level of vector competence is linked to both the exclusive focus on indoor vector collections and the long evolutionary history between *P. falciparum* and each of *An. gambiae* and *An. funestus*. Considering that the present study focused only on outdoor peridomestic collections of *Ny*. *darlingi* and on *P. vivax*, the observed range is unremarkable.

The negative correlation between mosquito infection rates and vector competence deserves a more detailed explanation. Note that, from Eq. (3), vector competence is the product of the per capita incidence rate (*Incidence/S*_*H*_) times the inverse of the HBR (*N*_*H*_*/*(*aN*_*M*_)) times the inverse of *P. vivax* prevalence in the mosquitoes (*N*_*M*_*/I*_*M*_). Therefore, for a given incidence level, for high values of HBR and high levels of *P. vivax* prevalence in the mosquitoes, the vector competence that would explain the observed variables would necessarily have low values. Conversely, for low values of HBR and low levels of *P. vivax* prevalence in the mosquitoes, vector competence that would explain the observed variables would necessarily have high values.

Vectorial capacity was calculated using Eq. (7) with inputs of the daily probability of one malaria case (estimated from incidence data using Eq. (5)) and the vector competence (from Eq. (3), above). The results are again of the same order of magnitude as those found by Dietz et al. [25, 26] for *P. falciparum* malaria in Nigeria. The maximum value these authors found was 21.74 in the village of Sugungum. However, they used a different formula for calculating vectorial capacity, the original Garret-Jones equation [24], which does not consider vector competence. That is, they computed vectorial capacity as the product of HBR by the factor *ap*^*n*^(− ln *p*), where *a* is the daily mosquito biting rate, *p* is the daily probability of mosquito survival and *n* is the mosquito mortality rate. When vector competence was included in the factor *ap*^*n*^(− ln *p*) (see [14] for a detailed discussion), the values obtained were very similar to the ones calculated. Therefore, the vectorial capacity values estimated for *Ny. darlingi* in transmitting *P. vivax* malaria are very high indeed.

Results of the estimation of the basic reproduction number (*R*_0_) showed values ranging from 3.3 to 58.7, obtained using Eq. (8), that is, the product of the vectorial capacity times a scaling factor comprised of the ratio between vector susceptibility and the infection and the recovery rate of humans from parasitemia, reflecting the variation in the vectorial capacity (Fig. 7). The maximum value of 58.7 means that one single human infected with *P. vivax*, in that specific location and time of data collection, was able to produce, throughout the mosquito population, almost 60 secondary malaria cases. This is an extremely high value, in the range of the estimations of *R*_0_ for malaria in Africa. More importantly, according to the reclassification of malaria endemicity proposed by Hay et al. [50], *P. vivax* endemicity was intense, stable and hyperendemic in Galo Velho settlement, Machadinho D’Oeste, and São Gabriel da Cachoeira. These values show the urgent need for implementing a combination of interventions for malaria control, that include insecticide-treated bed nets, indoor residual spraying when possible, and intensive treatment with anti-malarial drugs.

Both malaria transmission and its intensity are spatially and temporally heterogeneous in the Brazilian Amazon, including in the five municipalities in the present study. The biological, ecological and social processes that determine the effective dispersion of *P. vivax* propagation in a human community involve humans as hosts and a single vector species, *Ny*. *darlingi*. There are secondary vector species, but their presence in the outdoor and indoor human environments depends mostly on land use, presence of domestic vertebrates, and deforestation level [51, 52]. In the field collections carried out for this study, *Ny. darlingi* was the most abundant and the predominant species in the peridomestic area. Despite the constant presence of this species, the estimated vector competence, vectorial capacity and *R*_0_ showed variation among populations studied. The propagation and persistence of *Plasmodium* depends on the presence of hotspots for the pathogen and the capacity of the mosquito vector to disperse infectious bites in a human population with a distinct risk of being infected [9].

The estimated vector competence of *Ny. darlingi* to transmit *P. vivax* was low in the Brazilian Amazon region of Cruzeiro do Sul, Mâncio Lima, Lábrea, Machadinho D’Oeste and São Gabriel da Cachoeira. However, low vector competence appears to be offset by high biting rates and relatively high susceptibility to *Plasmodium* infection, as was demonstrated in experimental studies by Klein et al. [53] and other investigations reviewed by Hiwat and Bretas [32]. Moreover, female vector avoidance of defensive host behaviour by frequent feeding on lower legs and feet [34] might enhance the prevalence of *Plasmodium* infection in the vector and human populations. Plasticity in biting behaviour was observed in *Ny. darlingi* populations that shifted from biting mainly outdoors to more indoors in riverine communities near Iquitos, Peru, when the long-lasting insecticidal nets aged [38]. Such behaviour could increase the odds of *Ny. darlingi* exposure to an infectious human reservoir. Finally, low vector competence of *Ny*. *darlingi* for *P*. *vivax* infection may also be offset by increased survival in cases where preferred hosts are unavailable, and blood-feeding occurs on alternative hosts [37].

Other possible reasons for the combination of low vector competence and high *PvR*_0_ in the current study could include the highly heterogeneous HBR in human occupied forest landscapes. Such heterogeneity can be caused by deforestation level, presence of larval hotspots in the forest fringe, proximity of houses to the forest border, and human behaviour, all of which can increase mosquito–human contact. Furthermore, the field collections were conducted during the transition of the wet–dry season and in the dry season, when larval habitats have been shown to be more ecologically favourable to *Ny. darlingi*, thereby increasing female survival [54]. Consequently, the prevalence of *P. vivax* in the adult population is expected to be higher, as in [55].

## Conclusions

This is the first study to use mathematical modelling approach to calculate the vectorial capacity of *Ny. darlingi* employing field-collected data on malaria incidence in Brazil. Results of mathematical modelling approach to quantify entomological potential and transmission intensity of malaria bolster the importance of *Ny. darlingi* in the *P. vivax* malaria cycle in rural settlements in the Brazilian Amazon. The estimated low vector competence of this species seems to be offset by its relatively high vectorial capacity, and its occurrence in the peridomestic environment. These factors, in addition to poor housing conditions and human behaviour, resulted in very high *PvR*_0_, evidence for intense and stable transmission in the Galo Velho settlement, Machadinho D’Oeste, Rondônia and in São Gabriel da Cachoeira, Amazonas. Variation in vectorial capacity and *R*_0_ may be the result of heterogeneities in the level of human exposure to mosquito bites rather than variation in vector populations.

Strategic planning for malaria control employing interventions, such as mass screening for parasite reservoirs using rapid diagnostic test, and effective anti-malarial drugs treatments will decrease infection duration, local incidence, infection incidence, parasite reservoirs, and transmission intensity. In addition, indoor residual spray and long-lasting insecticide nets will decrease human exposure to mosquito bites to some degree, shrinking the transmission in these and other Amazonian municipalities with similar determinants of malaria risk. However, if the entomological potential either persists or increases, imported infections will replenish parasite reservoirs, boosting malaria transmission in areas that are receptive for *Plasmodium* propagation.

## Additional files

**Additional file 1.** Distribution of malaria according to epidemiological week (EPI) in five municipalities of the Amazonian states, 2014–2018, Brazil.

**Additional file 2.** The raw data of *Plasmodium vivax* malaria incidence in the five municipalities studied in Brazilian Amazon, and the parameters used in the fitting procedures are provided. These data do not include *Plasmodium falciparum* malaria.

## Abbreviations

API: annual parasite incidence
*b*_*t*_: vector competence
HLC: human landing catch
*h*_*t*_: the daily malaria incidence
*R*_0_: basic reproduction number
*V*_*Ct*_: vectorial capacity
SIVEP: Sistemas de Informações de Vigilância Epidemiológica da Malaria
