# Supplementary material for: Vector competence, vectorial capacity of Nyssorhynchus darlingi and the basic reproduction number of Plasmodium vivax in agricultural settlements in the Amazonian Region of Brazil
Source: Malar J. 2019 Apr 4;18:117. doi: 10.1186/s12936-019-2753-7 (PMC6449965; doi:10.1186/s12936-019-2753-7)
Supplement: Supplementary file 2 — Additional file 2. The raw data of Plasmodium vivax malaria incidence in the five municipalities studied in Brazilian Amazon, and the parameters used in the fitting procedures are provided. These data do not include Plasmodium falciparum malaria. [file 12936_2019_2753_MOESM2_ESM.docx]

**Additional file 2**

Vector Competence, Vectorial Capacity of *Nyssorhynchus darlingi* and the Basic Reproduction Number of *Plasmodium vivax* in Agricultural Settlements in Amazonian Brazil

Authors: Maria Anice M. Sallum, Jan E. Conn, Eduardo S. Bergo, Gabriel Z. Laporta, Leonardo S. M. Chaves, Sara A. Bickersmith, Tatiane M. P. de Oliveira, Elder Augusto G. Figueira, Gilberto Moresco, Lêuda Olívêr, Claudio J. Struchiner, Laith Yakob, Eduardo Massad

In additional file 2, it is provided the crude data of *Plasmodium vivax* malaria incidence in the five municipalities studied in Brazilian Amazon. Malaria incidence rates are distributed by epidemiological week and correspond to previous and month of field collections. In addition, it is included all parameters employed in the fitting procedures. The data do not include *Plasmodium falciparum* malaria or any other anopheline species in addition to *Ny. darlingi*.

In Brazil, morbidity and mortality of malaria have increased significantly during 2017-2018, with an 855% increase in *P. falciparum* malaria in some areas in São Gabriel da Cachoeira. From January 1st to November 30rd, 2018, 2% more cases were reported in Brazil. Whereas Mato Grosso, Pará, Roraima and Rondônia reported increase in malaria, Acre, Amazonas, Amapá, Maranhão and Tocantins state faced decrease in the transmission. For the current study, field-collections were carried out from 2015-2017, in rural settlements in five municipalities of three Amazonian states. Although all locations had a high API for malaria, the mathematical approach quantified differences in the entomological metrics and transmission potential. Hyperendemicity was detected in two regions, Galo Velho settlement in Machadinho D’Oeste, Rondônia and São Gabriel da Cachoeira, Amazonas.

**Malaria Hyperendemicity was detected in Machadinho D’Oeste (Rondônia state) and São Gabriel da Cachoeira (Amazonas state).**

Field-collections were carried out in a rural settlement located in the Machadinho River drainage catchment. Galo Velho settlement comprises a landscape mosaic, with forest fragments of various sizes interspersed with small rivers and streams, agriculture plantations, cattle farms, precarious human houses, areas that have been recently deforested and others in the process of clearing natural vegetation. Results of mathematical analyses showed high prevalence of *P. vivax* in *Ny. darlingi*, likely caused by the dynamic process of human occupation, ecological changes favoring the vector, and continuous human movement that facilitates the emergence / reemergence of *Plasmodium* in a receptive area. Determinants of malaria were investigated in the Machadinho rural settlement, Machadinho D’Oeste by Castro et al. [1, 2]. Accordingly, the complex interconnections among biological, ecological and sociodemographic systems determined vector abundance, degree of human-mosquito contact, propagation of *Plasmodium* in the area and the risk of acquiring malaria. Galo Velho (present study) and Machadinho settlements share similar local drivers of malaria, accounting for the high *P*. *vivax* infection rate detected in *Ny. darlingi*.

In Galo Velho settlement the interconnections among entomological and ecological factors determine high vector abundance, whereas sociodemographic factors enhance both local transmission and new introductions of *Plasmodium* favored by continuous human movement, high degree of human-mosquito contact because of human behavior, poor housing conditions, insufficient access to malaria commodities, leading to high prevalence of *P*. *vivax* infection in *Ny. darlingi*.

The burden of malaria hardly impacted local indigenous ethnical communities in the remote municipality of São Gabriel da Cachoeira, upper Rio Negro, northwestern Amazonas state. The estimated prevalence rate of *P. vivax* infection in *Ny. darlingi* was 2.95%, and the estimated *Ro* value indicates hyperendemicity of malaria in a level similar to those found for *P. falciparum* in sub-Saharan Africa countries. This municipality has the largest indigenous population in Brazil, comprising 23 ethnic groups, mainly Baré, Tukano and Baniwa. Malaria is endemic throughout the year, with two transmission peaks, one from January to March and the other from September to November. In 2017, the municipality recorded 12,144 cases of *P. vivax* and *P. falciparum* malaria, of which 835 were *P. falciparum*. The mean API of malaria overall was 252.8. Because of the geographical complexity of the region, with several remote communities accessible only by river, planning an effective malaria control program represents an enormous challenge for the municipality. Thus, alarmingly, malaria transmission increased by 50% between January and October 2018, with 13,671 reported cases of *P. vivax* and *P. falciparum* malaria, of which 3,765 were *P. falciparum*, an increase of 855% for *P. falciparum* in a single year. Malaria also increased in the town of São Gabriel da Cachoeira with the highest number of cases reported from the district of Parauari: 1,169 reported cases from January to October 2018 [3]. For the present study, field collections were restricted to periurban and rural areas that could be reached by road. Here, *Ny. darlingi* was abundant and the only species collected by HLC, including in two districts in the outskirts of the town of São Gabriel da Cachoeira - Tiago Montalvo and Miguel Quirino, where human occupation, deforestation and malaria are increasing.

The indigenous populations inhabiting areas along the river basin move from their lands to the town of São Gabriel approximately four times annually. This temporary back-and-forth movement was augmented by the federal cash transfer program, the "Bolsa Familia". This program is primarily focused on decreasing social and health inequalities of low-income families [4]. To obtain this Bolsa (cash income), indigenous families are obliged to travel long distances by river to reach São Gabriel da Cachoeira approximately four times annually, in the process being exposed for several consecutive nights to infectious bites of *Ny. darlingi*. Some become infected and subsequently propagate *Plasmodium* back to their communities, an additional complication for operational planning for malaria control. Rapid dispersal and intensification of malaria at the municipal level is evident from the staggering 855% increase in *P. falciparum* alone between 2017-2018. In addition, the number of localities reporting *P. falciparum* malaria jumped from 73/388 in 2017, to 237/488 in 2018. The 2.95% prevalence of *P. vivax* in *Ny. darlingi* detected in this study is likely underestimated considering the overall level of transmission in 2018.

| São Gabriel da Cachoeira – Fitting Malaria Incidence | | | | |
| --- | --- | --- | --- | --- |
|  | **Observed** | **Fitted** | | |
| Epidemiological Week | **Incidence** | **Mean** | **CI Inf** | **CI Sup** |
| 22 | **58** | **111** | **52** | **181** |
| 23 | **108** | **123** | **56** | **199** |
| 24 | **216** | **135** | **60** | **220** |
| 25 | **148** | **148** | **65** | **244** |
| 26 | **166** | **163** | **70** | **269** |
| 27 | **137** | **179** | **76** | **298** |
| 28 | **176** | **197** | **82** | **329** |
| 29 | **266** | **217** | **88** | **363** |
| 30 | **230** | **238** | **95** | **402** |
| 31 | **310** | **262** | **102** | **444** |
| 32 | **374** | **288** | **110** | **491** |
| 33 | **292** | **317** | **119** | **542** |
| 34 | **184** | **348** | **128** | **599** |
| 35 | **302** | **383** | **138** | **662** |
| 36 | **472** | **421** | **149** | **732** |
| 37 | **623** | **463** | **160** | **809** |
| 38 | **554** | **509** | **173** | **894** |
| 39 | **450** | **560** | **186** | **988** |
| 40 | **360** | **616** | **201** | **1092** |
| 41 | **868** | **677** | **216** | **1207** |

| **São Gabriel da Cachoeira - Fitting Parameter Estimates** | | | | | | | | |
| --- | --- | --- | --- | --- | --- | --- | --- | --- |
|  | **Parameter** | | **Estimate** | **Std. Error** | **95% Confidence Interval** | | **95% Trimmed Range** | |
|  |  |  |  |  | **Lower Bound** | **Upper Bound** | **Lower Bound** | **Upper Bound** |
| **Asymptotic** |  | **α** | **13.78** | **7.91** | **-2.85** | **30.41** |  |  |
|  |  | **β** | **0.09** | **0.02** | **0.06** | **0.13** |  |  |
| **Bootstrap** |  | **α** | **13.78*** | **12.71** | **-12.21** | **39.77** | **10.01*** | **20.08*** |
|  |  | **β** | **0.09*** | **0.02** | **0.05** | **0.14** | **0.08*** | **0.12*** |
| *values used in the fitting procedures | | | | | | | | |

| Cruzeiro do Sul - Fitting Incidence | | | | |
| --- | --- | --- | --- | --- |
|  | **Observed** | **Fitted** | | |
| Epidemiological Week | **Incidence** | **Mean** | **CI Inf** | **CI Sup** |
| 12 | **470** | **639** | **478** | **891** |
| 13 | **504** | **647** | **480** | **908** |
| 14 | **809** | **656** | **482** | **925** |
| 15 | **711** | **664** | **485** | **943** |
| 16 | **874** | **673** | **487** | **961** |
| 17 | **666** | **682** | **490** | **979** |
| 18 | **790** | **691** | **492** | **998** |
| 19 | **669** | **700** | **495** | **1017** |
| 20 | **773** | **709** | **497** | **1037** |
| 21 | **756** | **718** | **500** | **1057** |
| 22 | **742** | **728** | **502** | **1077** |
| 23 | **806** | **737** | **505** | **1098** |
| 24 | **742** | **747** | **507** | **1119** |
| 25 | **815** | **756** | **510** | **1140** |
| 26 | **672** | **766** | **512** | **1162** |
| 27 | **602** | **776** | **515** | **1184** |
| 28 | **753** | **787** | **517** | **1207** |
| 29 | **753** | **797** | **520** | **1230** |
| 30 | **826** | **807** | **523** | **1254** |
| 31 | **694** | **818** | **525** | **1278** |
| 32 | **823** | **829** | **528** | **1302** |
| 33 | **904** | **839** | **531** | **1327** |
| 34 | **935** | **850** | **533** | **1353** |
| 35 | **1000** | **862** | **536** | **1379** |
| 36 | **910** | **873** | **539** | **1405** |
| 37 | **818** | **884** | **541** | **1432** |
| 38 | **809** | **896** | **544** | **1460** |
| 39 | **941** | **908** | **547** | **1488** |
| 40 | **1042** | **919** | **549** | **1516** |

| **Cruzeiro do Sul - Fitting Parameter Estimates** | | | | | | | | |
| --- | --- | --- | --- | --- | --- | --- | --- | --- |
|  | **Parameter** | | **Estimate** | **Std. Error** | **95% Confidence Interval** | | **95% Trimmed Range** | |
|  |  |  |  |  | **Lower Bound** | **Upper Bound** | **Lower Bound** | **Upper Bound** |
| **Asymptotic** |  | **α** | **546.589** | **44.381** | **455.526** | **637.651** |  |  |
|  |  | **β** | **.013** | **.003** | **.008** | **.019** |  |  |
| **Bootstrap** |  | **α** | **546.589*** | **58.809** | **426.310** | **666.867** | **449.846*** | **709.036*** |
|  |  | **β** | **.013*** | **.003** | **.007** | **.020** | **.005*** | **.019*** |
| *values used in the fitting procedures | | | | | | | | |

| **Mâncio Lima - Fitting Incidence** | | | | |
| --- | --- | --- | --- | --- |
|  | **Observed** | **Fitted** | | |
| **Epidemiological Week** | **Incidence** | **Mean** | **CI Inf** | **CI Sup** |
| **14** | **213** | **219** | **156** | **313** |
| **15** | **242** | **226** | **159** | **324** |
| **16** | **284** | **232** | **162** | **335** |
| **17** | **242** | **239** | **165** | **346** |
| **18** | **277** | **245** | **168** | **357** |
| **19** | **203** | **252** | **171** | **369** |
| **20** | **188** | **260** | **174** | **382** |
| **21** | **192** | **267** | **177** | **395** |
| **22** | **274** | **274** | **180** | **408** |
| **23** | **231** | **282** | **183** | **421** |
| **24** | **363** | **290** | **187** | **436** |
| **25** | **274** | **299** | **190** | **450** |
| **26** | **210** | **307** | **194** | **465** |
| **27** | **295** | **316** | **197** | **481** |
| **28** | **316** | **325** | **201** | **497** |
| **29** | **370** | **334** | **204** | **514** |
| **30** | **341** | **343** | **208** | **531** |
| **31** | **352** | **353** | **212** | **549** |
| **32** | **476** | **363** | **216** | **567** |
| **33** | **459** | **374** | **220** | **586** |
| **34** | **270** | **384** | **224** | **606** |
| **35** | **622** | **395** | **228** | **626** |
| **36** | **501** | **406** | **232** | **647** |
| **37** | **409** | **418** | **236** | **669** |
| **38** | **437** | **430** | **240** | **691** |
| **39** | **491** | **442** | **245** | **715** |
| **40** | **540** | **454** | **249** | **739** |
| **41** | **494** | **467** | **254** | **763** |
| **42** | **462** | **481** | **258** | **789** |
| **43** | **377** | **494** | **263** | **815** |
| **44** | **519** | **508** | **268** | **843** |
| **45** | **427** | **523** | **273** | **871** |
| **46** | **359** | **537** | **278** | **900** |
| **47** | **352** | **553** | **283** | **931** |
| **48** | **611** | **568** | **288** | **962** |
| **49** | **626** | **585** | **293** | **994** |
| **50** | **711** | **601** | **298** | **1027** |
| **51** | **725** | **618** | **304** | **1062** |

| **Mâncio Lima - Fitting Parameter Estimates** | | | | | | | | |
| --- | --- | --- | --- | --- | --- | --- | --- | --- |
|  | **Parameter** | | **Estimate** | **Std. Error** | **95% Confidence Interval** | | **95% Trimmed Range** | |
|  |  |  |  |  | **Lower Bound** | **Upper Bound** | **Lower Bound** | **Upper Bound** |
| **Asymptotic** |  | **α** | **148.253** | **20.610** | **106.454** | **190.052** |  |  |
|  |  | **β** | **.028** | **.003** | **.021** | **.035** |  |  |
| **Bootstrap** |  | **α** | **148.253*** | **18.597** | **110.217** | **186.290** | **121.264*** | **197.303*** |
|  |  | **β** | **.028*** | **.004** | **.021** | **.036** | **.018*** | **.033*** |
| *values used in the fitting procedures | | | | | | | | |

| **Machadinho D'Oeste - Fitting Incidence** | | | | |
| --- | --- | --- | --- | --- |
|  | **Observed** | **Fitted** | | |
| **Epidemiological Week** | **Incidence** | **Mean** | **CI Inf** | **CI Sup** |
| **1** | **53** | **27** | **14** | **47** |
| **2** | **27** | **29** | **15** | **52** |
| **3** | **13** | **31** | **15** | **57** |
| **4** | **27** | **34** | **16** | **62** |
| **5** | **67** | **37** | **17** | **68** |
| **6** | **53** | **40** | **18** | **74** |
| **7** | **93** | **44** | **19** | **82** |
| **8** | **67** | **47** | **20** | **89** |
| **9** | **53** | **52** | **21** | **98** |
| **10** | **27** | **56** | **22** | **107** |
| **11** | **120** | **61** | **24** | **117** |
| **12** | **146** | **66** | **25** | **128** |
| **13** | **67** | **72** | **26** | **140** |
| **14** | **13** | **78** | **28** | **153** |
| **15** | **106** | **85** | **29** | **167** |
| **16** | **146** | **92** | **31** | **183** |
| **17** | **80** | **100** | **33** | **200** |
| **18** | **53** | **109** | **34** | **219** |
| **19** | **133** | **118** | **36** | **240** |
| **20** | **106** | **129** | **38** | **263** |
| **21** | **226** | **140** | **40** | **287** |
| **22** | **93** | **152** | **42** | **314** |
| **23** | **239** | **165** | **45** | **344** |
| **24** | **133** | **179** | **47** | **376** |
| **25** | **186** | **195** | **50** | **412** |
| **26** | **226** | **212** | **52** | **451** |
| **27** | **160** | **230** | **55** | **493** |
| **28** | **120** | **250** | **58** | **539** |
| **29** | **160** | **271** | **61** | **590** |
| **30** | **346** | **295** | **65** | **646** |
| **31** | **372** | **320** | **68** | **707** |
| **32** | **439** | **348** | **72** | **773** |

| **Machadinho D’Oeste - Fitting Parameter Estimates** | | | | | | | | |
| --- | --- | --- | --- | --- | --- | --- | --- | --- |
|  | **Parameter** | | **Estimate** | **Std. Error** | **95% Confidence Interval** | | **95% Trimmed Range** | |
|  |  |  |  |  | **Lower Bound** | **Upper Bound** | **Lower Bound** | **Upper Bound** |
| **Asymptotic** |  | **α** | **24.451** | **7.535** | **9.063** | **39.839** |  |  |
|  |  | **β** | **.083** | **.011** | **.060** | **.0106** |  |  |
| **Bootstrap** |  | **α** | **24.451*** | **9.119** | **5.802** | **43.101** | **13.206*** | **43.407*** |
|  |  | **β** | **.083*** | **.015** | **.052** | **.0114** | **.053*** | **.900*** |
| *values used in the fitting procedures | | | | | | | | |

| **Lábrea - Fitting Incidence** | | | | |
| --- | --- | --- | --- | --- |
|  | **Observed** | **Fitted** | | |
| **Epidemiological Week** | **Incidence** | **Mean** | **CI Inf** | **CI Sup** |
| **1** | **256** | **261** | **220** | **310** |
| **2** | **256** | **271** | **226** | **323** |
| **3** | **364** | **280** | **232** | **337** |
| **4** | **312** | **290** | **239** | **351** |
| **5** | **288** | **301** | **245** | **365** |
| **6** | **248** | **311** | **252** | **381** |
| **7** | **284** | **322** | **259** | **397** |
| **8** | **276** | **334** | **266** | **413** |
| **9** | **220** | **346** | **273** | **430** |
| **10** | **280** | **358** | **281** | **448** |
| **11** | **521** | **371** | **288** | **467** |
| **12** | **344** | **384** | **296** | **487** |
| **13** | **336** | **398** | **304** | **507** |
| **14** | **328** | **412** | **313** | **528** |
| **15** | **533** | **427** | **321** | **550** |
| **16** | **525** | **442** | **330** | **574** |
| **17** | **517** | **457** | **339** | **598** |
| **18** | **657** | **474** | **348** | **623** |
| **19** | **605** | **491** | **358** | **649** |
| **20** | **557** | **508** | **368** | **676** |
| **21** | **505** | **526** | **378** | **704** |
| **22** | **513** | **545** | **388** | **733** |
| **23** | **488** | **564** | **399** | **764** |
| **24** | **581** | **584** | **410** | **796** |
| **25** | **484** | **605** | **421** | **829** |
| **26** | **488** | **627** | **432** | **864** |
| **27** | **589** | **649** | **444** | **900** |
| **28** | **717** | **672** | **456** | **938** |
| **29** | **805** | **696** | **469** | **977** |

| **Lábrea - Fitting Parameter Estimates** | | | | | | | | |
| --- | --- | --- | --- | --- | --- | --- | --- | --- |
|  | **Parameter** | | **Estimate** | **Std. Error** | **95% Confidence Interval** | | **95% Trimmed Range** | |
|  |  |  |  |  | **Lower Bound** | **Upper Bound** | **Lower Bound** | **Upper Bound** |
| **Asymptotic** |  | **α** | **252.320** | **24.300** | **202.460** | **302.180** |  |  |
|  |  | **β** | **.035** | **.005** | **.026** | **.044** |  |  |
| **Bootstrap** |  | **α** | **252.320*** | **19.769** | **211.887** | **292.753** | **214.223*** | **297.610*** |
|  |  | **β** | **.035*** | **.003** | **.028** | **.042** | **.027*** | **.041*** |
| *values used in the fitting procedures | | | | | | | | |

References

1. Castro MC de, Sawyer DO, Singer BH. Spatial patterns of malaria in the Amazon: implications for surveillance and targeted interventions. Health Place. 2007;13(2):368–380.
2. Castro M C de, Monte-Mór RL, Sawyer DO, Singer BH. Malaria risk on the Amazon frontier. Proc Natl Acad Sci U S A. 2006;103(7):2452–2457.
3. Ministério da Saúde do Brasil. Datasus. Sivep Malária. Sistema Eletrônico do Serviço de Informações ao Cidadão (e-SIC).https://esic.cgu.gov.br/sistema/site/index.aspx. 2018.

Alves H, Escorel S. Social exclusion and health inequity: a case study based on a cash distribution program (Bolsa Família) in Brazil. Rev Panam Salud Publica. 2013;34(6):429–436.
